# A randomized trial of icosapent ethyl in ambulatory patients with COVID-19

**DOI:** 10.1016/j.isci.2021.103040

**Published:** 2021-08-26

**Authors:** Andrew Kosmopoulos, Deepak L. Bhatt, Gus Meglis, Raj Verma, Yi Pan, Adrian Quan, Hwee Teoh, Maya Verma, Lixia Jiao, Robert Wang, Rebecca A. Juliano, Mahesh Kajil, Mikhail N. Kosiborod, Basel Bari, Abdullahi A. Berih, Mallory Aguilar, Antonnette Escano, Andrew Leung, Idelta Coelho, Makoto Hibino, Rafael Díaz, R. Preston Mason, Ph. Gabriel Steg, Tabassome Simon, Alan S. Go, Andrew P. Ambrosy, Richard Choi, Arthur M. Kushner, Lawrence A. Leiter, Mohammed Al-Omran, Subodh Verma, C. David Mazer

**Affiliations:** 1 North York Diagnostic and Cardiac Centre, Toronto, ON, Canada; 2 Division of Cardiac Surgery, Li Ka Shing Knowledge Institute of St. Michael’s Hospital, Unity Health Toronto, Toronto, ON, Canada; 3 Department of Pharmacology & Toxicology, University of Toronto, Toronto, ON, Canada; 4 Brigham and Women’s Hospital Heart and Vascular Center, Harvard Medical School, 75 Francis St., Boston, MA 02115, USA; 5 Division of Endocrinology and Metabolism, Li Ka Shing Knowledge Institute of St. Michael’s Hospital, Unity Health Toronto, Toronto, ON, Canada; 6 Amarin Pharma Inc., Bridgewater, NJ, USA; 7 Department of Cardiology, Saint Luke’s Mid America Heart Institute, Kansas City, MO, USA; 8 Department of Medicine, University of Missouri-Kansas City, Missouri, USA; 9 The George Institute for Global Health, Sydney, NSW, Australia; 10 University of New South Wales, Sydney, NSW, Australia; 11 Markham Health+Plex Medical Centre, Markham, ON, Canada; 12 Humber Centre for Family Health, Etobicoke, ON, Canada; 13 Alpha Laboratories Inc., Toronto, ON, Canada; 14 Estudios Clínicos Latino América, Instituto Cardiovascular de Rosario, Rosario, Argentina; 15 Cardiovascular Division, Brigham and Women's Hospital, Harvard Medical School, Boston, MA, USA; 16 Université de Paris, Assistance Publique-Hôpitaux de Paris, INSERM 1148, Paris, France; 17 French Alliance for Cardiovascular Trials (FACT), Paris, France; 18 National Heart & Lung Institute NHLI, Imperial College, Royal Brompton Hospital, London, UK; 19 Department of Clinical Pharmacology, Unité de Recherche Clinique (URCEST), Assistance Publique-Hôpitaux de Paris, Hôpital Saint Antoine, Sorbonne Université, site St Antoine, INSERM U-698, Paris, France; 20 Division of Research, Kaiser Permanente Northern California, Oakland, CA, USA; 21 Departments of Epidemiology, Biostatistics and Medicine, University of California at San Francisco, San Francisco, CA, USA; 22 Division of Nephrology, Department of Medicine, Stanford University School of Medicine, Stanford, CA, USA; 23 Division of Cardiology, Kaiser Permanente San Francisco Medical Center, San Francisco, CA, USA; 24 Riverside Cardiology and Diagnostic Imaging; Division of Cardiology, St. Joseph's Health Centre, Unity Health Toronto, Toronto, ON, Canada; 25 Humber River Hospital, Toronto, ON, Canada; 26 Department of Medicine, University of Toronto, Toronto, ON, Canada; 27 Department of Nutritional Sciences, University of Toronto, Toronto, ON, Canada; 28 Division of Vascular Surgery, Li Ka Shing Knowledge Institute of St. Michael’s Hospital, Unity Health Toronto, Toronto, ON, Canada; 29 Department of Surgery, University of Toronto, Toronto, ON, Canada; 30 Canadian Medical and Surgical Knowledge Translation Research Group, Toronto, ON, Canada; 31 Department of Anesthesia, Li Ka Shing Knowledge Institute of St. Michael’s Hospital, Unity Health Toronto, Toronto, ON, Canada; 32 Department of Anesthesiology and Pain Medicine, University of Toronto, Toronto, ON, Canada; 33 Department of Physiology, University of Toronto, Toronto, ON, Canada

**Keywords:** Health sciences, Medicine

## Abstract

The coronavirus disease 2019 (COVID-19) pandemic remains a source of considerable morbidity and mortality throughout the world. Therapeutic options to reduce symptoms, inflammatory response, or disease progression are limited. This randomized open-label trial enrolled 100 ambulatory patients with symptomatic COVID-19 in Toronto, Canada. Results indicate that icosapent ethyl (8 g daily for 3 days followed by 4 g daily for 11 days) significantly reduced high-sensitivity C-reactive protein (hs-CRP) and improved symptomatology compared with patients assigned to usual care. Specifically, the primary biomarker endpoint, change in hs-CRP, was significantly reduced by 25% among treated patients (−0.5 mg/L, interquartile range [IQR] [−6.9,0.4], within-group p = 0.011). Conversely, a non-significant 5.6% reduction was observed among usual care patients (−0.1 mg/L, IQR [−3.2,1.7], within-group p = 0.51). An unadjusted between-group primary biomarker analysis was non-significant (p = 0.082). Overall, this report provides evidence of an early anti-inflammatory effect of icosapent ethyl in a modest sample, including an initial well-tolerated loading dose, in symptomatic outpatients with COVID-19. ClinicalTrials.gov Identifier: NCT04412018.

## Highlights

• hs-CRP was significantly reduced within the icosapent ethyl cohort (p value = 0.011)
• Total symptom prevalence was significantly reduced in treatment versus usual care
• Treated participants had significant FLU-PRO score reductions versus usual care
• First evidence of a well-tolerated icosapent ethyl loading dose (8 g/day for 3 days)

## Graphical abstract

**Figure:**
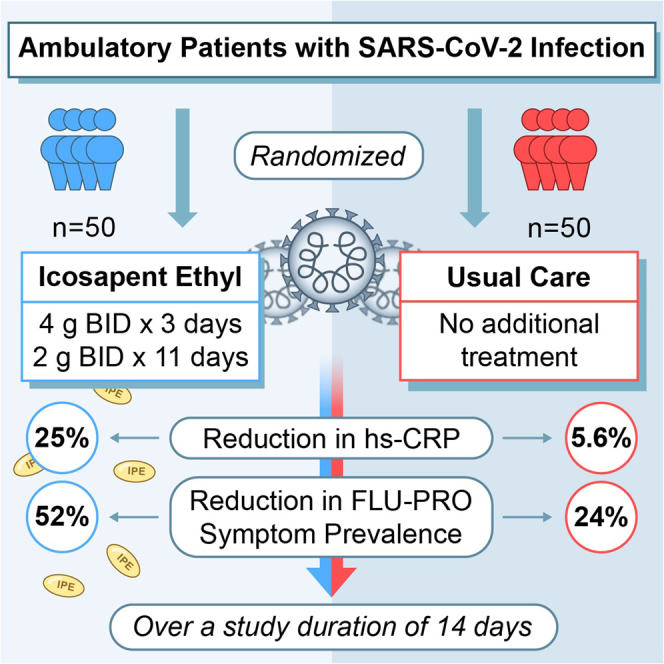

## Introduction

The coronavirus disease 2019 (COVID-19) pandemic remains a source of considerable morbidity and mortality throughout the world, with few safe and effective treatments currently available. Furthermore, the therapies that have demonstrated efficacy (e.g., corticosteroids) studied hospitalized and/or markedly ill patients (Horby et al., 2021). For the majority of patients in the community who develop symptomatic COVID-19, therapeutic options to reduce symptoms, inflammatory response, or disease progression are limited.

Icosapent ethyl (IPE) is a highly purified ethyl ester of eicosapentaenoic acid (EPA) that is available by prescription in the United States and Canada. In the recently completed REDUCE-IT trial, IPE (administered at an oral dose of 4 g daily) reduced major adverse cardiovascular events by 25% in people with or at risk of cardiovascular disease, in a median follow-up period of 4.9 years (Bhatt et al., 2019a). IPE was also associated with a 20% reduction in cardiovascular death. Further analyses revealed that IPE also reduced recurrent ischemic events, including the need for first and subsequent revascularization procedures (Bhatt et al., 2019b; Peterson et al., 2021).

Mechanistically, IPE is believed to afford potent vasculoprotective effects (Mason et al., 2020). Several in vitro and in vivo studies have suggested that EPA improves endothelial function, limits vascular inflammation and reactive oxygen species production, reduces vascular thrombosis, and impedes multiple processes involved in aberrant vascular repair/remodeling (Budoff et al., 2020; Mason et al., 2020; Wang et al., 2020). Accumulating data also point toward a potent anti-inflammatory effect of EPA, mediated in part through the synthesis of specialized pro-resolving mediators (Bhatt et al., 2020a; Serhan and Levy, 2018). These mediators are powerful modulators of the innate and adaptive immune system, implicated in overall resolution of inflammation through effects on host defense, neutrophil trafficking, and cytokine/chemokine elaboration. EPA has been shown to have bactericidal properties and also to limit viral replication and specifically, protectin D1 (PD1), a lipid mediator derived from docosahexaenoic acid (DHA), markedly reduced influenza A virus replication, and decreased lethal influenza events in mice (Bhatt et al., 2020b; Morita et al., 2013). EPA also assists to reduce systemic circulating markers of inflammation in humans (including high-sensitivity C-reactive protein [hs-CRP] and interleukin-6, among others) with most of the evidence coming from patients with hypertriglyceridemia and increased circulating anti-inflammatory and pro-resolving lipid mediators (Lamon-Fava et al., 2021; Mason, 2019; Muhammad et al., 2011).

Several lines of evidence implicate endothelial dysfunction as a critical mediator and/or transducer of COVID-19-related pathobiology (Perico et al., 2021; Teuwen et al., 2020). It is currently believed that SARS-CoV-2 binds endothelial ACE2 to promote widespread endothelialitis, characterized by heightened endothelial-immune interactions and thromboinflammation.

Clinically, omega-3 fatty acid use was explored as an agent against COVID-19 in a recent trial by Doaei et al. (2021), evaluating a 1000 mg daily concoction (comprised of 400 mg EPA, 200 mg DHA) over a two-week follow-up period. The authors found a significant increase in one-month survival rate, improved kidney function, and effects on certain arterial blood gas parameters, favoring treatment over control groups (Doaei et al., 2021).

Given the aforementioned clinical and biological effects of marine omega-3 fatty acids, we hypothesized that IPE may serve as a potential therapy to reduce inflammation and improve symptoms in patients with COVID-19 (Regidor et al., 2020). We herein report the primary results of the open-label, randomized VASCEPA-COVID-19 CardioLink-9 trial of IPE compared with usual care in ambulatory patients with COVID-19 (ClinicalTrials.gov Identifier: NCT04412018).

## Results and discussion

With ethical committee approval and informed consent, patients within the Greater Toronto Area, Canada, were recruited if they received a positive local SARS-CoV-2 polymerase chain reaction (PCR) test result within the preceding 72 hr of enrollment and at least one of the following symptoms: fever, cough, sore throat, shortness of breath, or myalgia. Individuals were excluded if they were hospitalized, pregnant, had a history of acute (<1 month) end-organ injury (e.g., myocardial infarction, stroke, hospitalization for acute lung, liver, or kidney disease), history of acute or chronic pancreatitis, active severe liver disease, hypersensitive to fish, shellfish, or ingredients of IPE, history of hemodynamic instability within the past 72 hr, or other situations that reduced the likelihood of completing the study protocol. There was no requirement for having hypertriglyceridemia at entry.

### Study population

**Figure 1:**
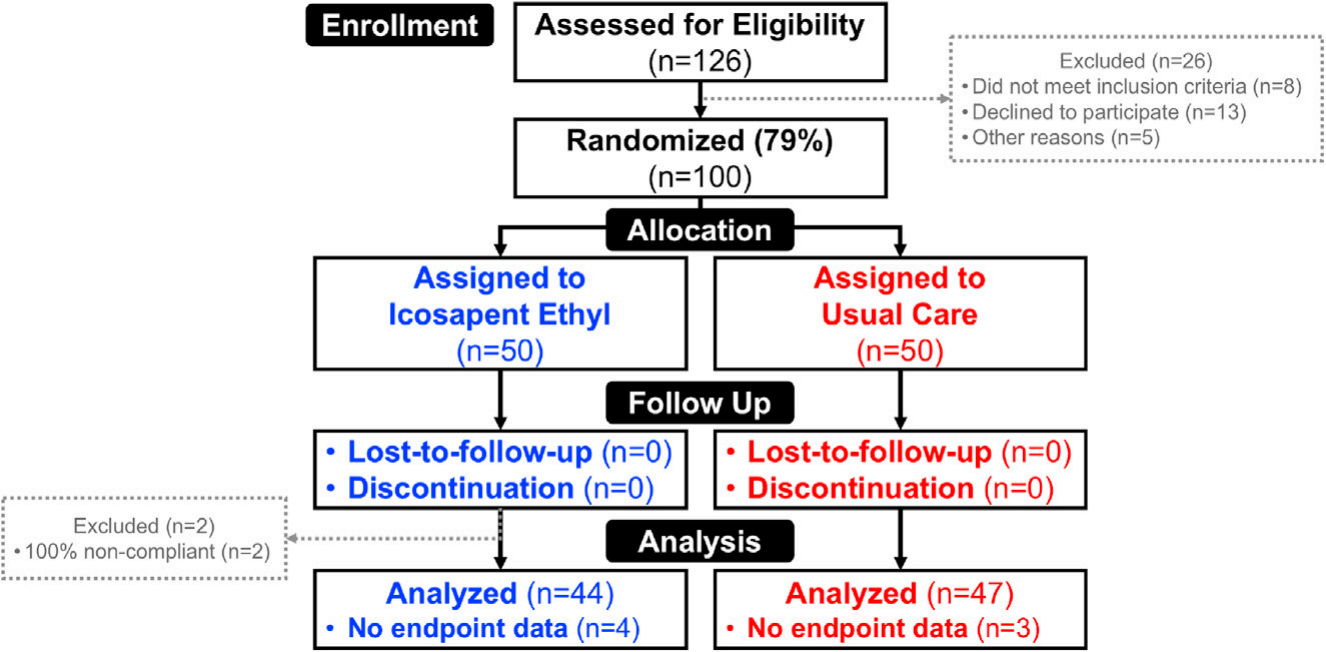
Study CONSORT diagram A schematic outlining the number of individuals who were assessed for eligibility, randomized, and included in the analyses. Thirteen people declined to participate. Approximately, 79% of individuals assessed for eligibility consented to participate and were subsequently randomized. Of the 50 individuals randomized to each group, data from 12% (n = 6) of the IPE group and 6% (n = 3) of the usual care group were not analyzed for the primary biomarker endpoint based on the lack of paired biomarker data.

**Figure 2:**
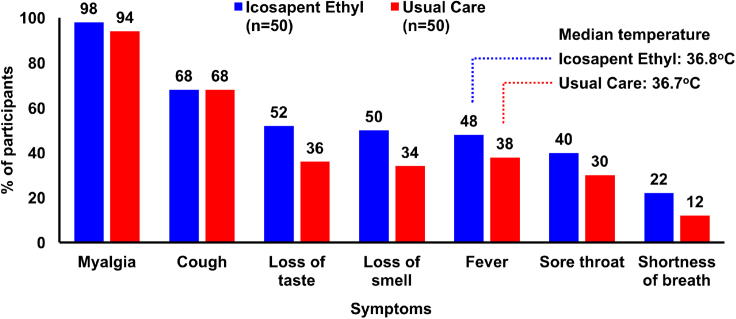
Baseline symptoms COVID-19 symptoms within 72 hr preceding the baseline visit. Although the IPE group was slightly more symptomatic, the entire study population presented with a large number of baseline symptoms. Almost all patients (98% and 94% in the IPE group and usual care group, respectively) had myalgia. Over half of individuals in both groups had the presence of a cough. Within the IPE arm, 50 ± 2% of patients experienced loss of taste, loss of smell, or a fever. These symptoms were present in approximately one-third of the usual care arm. The remaining categories (sore throat and shortness of breath) displayed not more than a 40% prevalence in either group. All between-group comparisons were non-significant. Data shown are for the intention-to-treat population.

Among 126 individuals assessed for eligibility, 79.4% (n = 100) of them were randomized in a 1:1 ratio to either IPE (a loading dose of 4 g twice daily taken orally for 3 days, followed by 2 g twice daily for 11 days) or usual care (see STAR Methods and Figure 1). Enrollment began on June 4, 2020, with 14+3-day follow-up through November 6, 2020. Baseline characteristics were similar between groups, and all patients were symptomatic, with myalgia, cough, and loss of taste/smell being among the most common symptoms (Figure 2 and Table S1).

### Prespecified primary biomarker endpoint and post hoc secondary biomarker analyses

The hs-CRP level is hypothesized to assist in predicting COVID-19-induced respiratory decline (Mueller et al., 2020). The prespecified primary biomarker outcome was unadjusted change in hs-CRP from baseline (day 1) to follow-up (day 14 + 3). Median between-group baseline levels of hs-CRP were not statistically different (IPE group, 3.2 mg/L; interquartile range [IQR = 0.9, 11.6]; usual care group, 2.3 mg/L [IQR = 0.7, 6.5], p = 0.16). Among patients randomized to receive IPE, the primary biomarker endpoint of median hs-CRP change at follow-up was −0.5 mg/L (IQR [−6.9, 0.4], p = 0.011), which corresponds to a 25% significant reduction from baseline levels (Table 1). Among those assigned to receive usual care, median hs-CRP change at follow-up was −0.1 mg/L (IQR [−3.2, 1.7], p = 0.51). However, a non-significant p value of 0.082 was obtained when comparing the two groups for unadjusted values. In a post hoc analysis, the between-group difference was significant (p = 0.043) after adjustment for age, sex and baseline predicted cardiovascular risk (Table 1). The model adjustments for baseline covariates post hoc were performed based on the current literature (O’Driscoll et al., 2020).

**Table 1:** Prespecified (unadjusted) and post hoc (adjusted) primary biomarker endpoint analyses: change in hs-CRP from baseline to follow-up

hs-CRP Median (IQR)	Baseline (mg/L)	14 + 3 Days (mg/L)	Median percent change from baseline	Median change from baseline (mg/L)	p value^a^
Icosapent ethyl (n = 44)	3.2 (0.9, 11.6)	1.6 (0.6, 4.4)	−25.0 (−80.1, 26.7)	−0.5 (−6.9, 0.4)	0.011
Usual care (n = 47)	2.3 (0.7, 6.5)	2.1 (0.5, 5.8)	−5.6 (−57.1, 84.2)	−0.1 (−3.2, 1.7)	0.51

Within-group comparisons of hs-CRP levels for the IPE and usual care cohorts. A significant relative reduction of 25% was observed in the hs-CRP level (median change from baseline of −0.5, p = 0.011) in the IPE cohort while there were no significant changes in the usual care cohort (median change from baseline of −0.1, p = 0.51). The between-group difference was not significant for the unadjusted values (p = 0.082 for change from baseline hs-CRP), but after post hoc adjustment for age, sex, and baseline cardiovascular risk, the between-group p value was significant (p = 0.043 for change from baseline hs-CRP). Sex and age adjustments: men <45 versus ≥45 years and women <55 versus ≥55 years. Adjustments were warranted based on current literature and occurred post hoc (see STAR Methods section) (O’Driscoll et al., 2020). Baseline cardiovascular risk is described as the absence or presence of cardiovascular comorbidities. hs-CRP, high-sensitivity C-reactive protein; IQR, interquartile range.

a p value (within-group median change from baseline); p value (between-group, unadjusted, prespecified) = 0.082; p value (between-group, adjusted, post hoc) = 0.043.

Additionally, post hoc analyses of secondary biomarkers revealed a reduction in D-dimer levels from baseline to follow-up within the IPE group (Table S2). Secondary laboratory parameters are shown in Table S3.

### Prespecified primary clinical endpoint and FLU-PRO symptom prevalence

The prespecified primary clinical outcome for the trial was the change in symptomatology as assessed by the InFLUenza Patient-Reported Outcome (FLU-PRO) score, a validated patient-reported outcome measure used to evaluate the presence, severity, and duration of flu-like symptoms in clinical trials (Han et al., 2018; Powers et al., 2018). The 32-item FLU-PRO score provides a comprehensive evaluation of the full range of symptoms across six symptom domains including nose, throat, eyes, chest/respiratory, gastrointestinal, and body/systemic, and the questionnaire was adapted to also capture COVID-19-specific symptoms such as loss of taste/smell. Patients were asked to rate each FLU-PRO domain on a 5-point ordinal scale that ranged from 0 (no symptoms) to 4 (very frequent symptoms) and to answer COVID-19-specific questions with yes/no (see STAR Methods).

**Figure 3:**
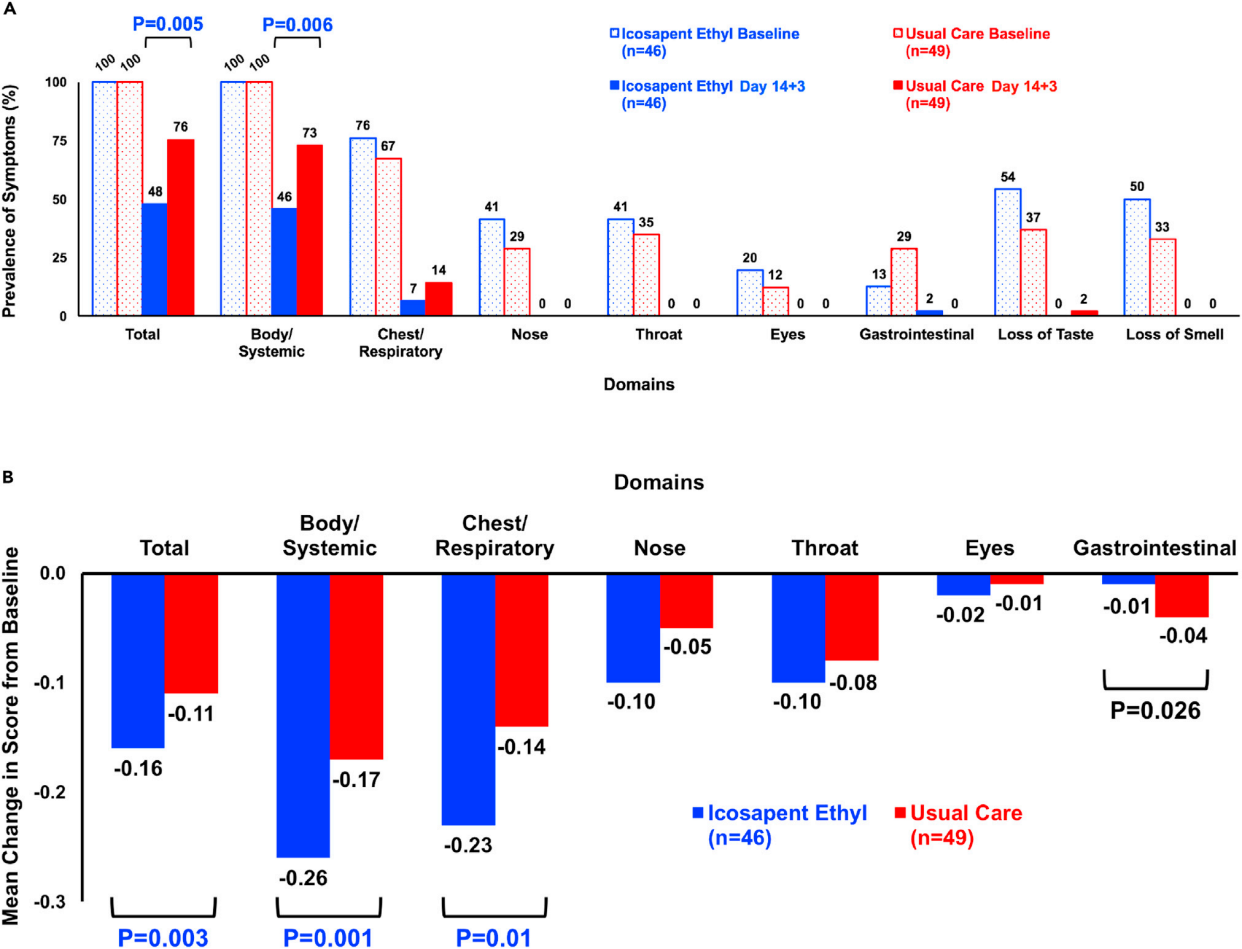
Change in total and individual domain FLU-PRO symptom prevalence and scores (A) FLU-PRO and COVID-19-specific symptom prevalence. The prevalence of FLU-PRO and COVID-19-specific (loss of taste, loss of smell) symptoms at baseline and follow-up distributed by total and individual domains. A significant reduction in the total (p = 0.005) and body/systemic (p = 0.006) domains occurred between groups. A numerically larger reduction in chest/respiratory symptoms occurred in the IPE (69%) compared with the usual care cohort (53%), although not a significant difference. The remaining domains had similar reductions in prevalence between groups. Values shown are based on the number of patients with complete paired data and non-zero treatment compliance. All domains within the IPE and usual care cohorts had significant within-group differences, comparing the number of symptomatic patients at baseline versus follow-up, via Chi-square analyses: IPE (p < 0.01 within all domains except the gastrointestinal domain [p = 0.049]); usual care (p ≤ 0.01 within all domains). (B) Prespecified primary clinical endpoint - change in FLU-PRO scores. Mean change in FLU-PRO scores from baseline to follow-up, distributed by total and individual domains. A significant score reduction in the IPE group compared with the usual care cohort occurred in the total (p = 0.003), body/systemic (p = 0.001), and chest/respiratory (p = 0.01) domains. A significant score reduction in the usual care group compared with the IPE cohort occurred in the gastrointestinal domain (p = 0.026). The remaining domains had non-significant changes in scores between groups. Values shown are based on the number of patients with complete paired data and non-zero treatment compliance. Mean FLU-PRO scores and associated between-group p values at baseline, per domain: Total: IPE = 0.18, usual care = 0.14, p = 0.03; body/systemic: IPE = 0.28, usual care = 0.23, p = 0.04; gastrointestinal: IPE = 0.01, usual care = 0.04, p = 0.04; chest/respiratory: IPE = 0.25, usual care = 0.16, p = 0.03; eyes: IPE = 0.02, usual care = 0.01, p = 0.34; throat; IPE = 0.10, usual care = 0.08, p = 0.59; nose: IPE = 0.10, usual care = 0.05, p = 0.16.

By design, at entry, the prevalence of ≥1 FLU-PRO symptom was 100% in both groups (Figure 3A). At 14+3-day follow-up, total symptom prevalence was reduced by 52% in the IPE group (p < 0.0001) and by 24% within the usual care group (p = 0.0002). The reductions in symptom prevalence were significant between groups (p = 0.005). At baseline, 100% of patients had body/systemic symptoms, the prevalence of which was markedly attenuated in those allocated to IPE versus usual care (p = 0.006) (Figure 3A).

### Additional post hoc analyses

We next examined the clinical endpoint of change in FLU-PRO total and domain-specific scores from baseline to follow-up, post hoc. Among IPE-treated patients with non-zero treatment compliance, the change in mean FLU-PRO scores in the total domain (−0.16, SD [0.09], p < 0.0001) and all subsequent domains except gastrointestinal were significant. Within the usual care cohort, the change in mean FLU-PRO scores in the total domain (−0.11, standard deviation, SD [0.08], p < 0.0001) and all subsequent domains were significant. Notably, score reductions in IPE-assigned patients were larger in magnitude compared to usual care patients in all domains except gastrointestinal. Significant between-group differences in FLU-PRO total (p = 0.003), body/systemic (p = 0.001), and chest/respiratory (p = 0.01) domain scores were found in favor of patients randomized to IPE over usual care (Figure 3B).

Subsequently, we examined if a change in hs-CRP levels correlated with a clinical reduction in FLU-PRO symptoms. These post hoc analyses demonstrated significant fair-to-moderate-sized correlations between a decrease in hs-CRP levels and improvement in FLU-PRO symptoms (score reduction) within the IPE group for total (p = 0.005), body/systemic (p = 0.006), and chest/respiratory (p < 0.001) domains, with no significant correlations observed in the usual care group (Table S4).

### Reported adverse events

Treatment with IPE was well tolerated with a low rate of adverse events overall and a small numeric excess of gastrointestinal side effects (Table S5).

### Conclusions

In summary, we report possible evidence of an early anti-inflammatory effect of IPE, including an initial loading dose, in symptomatic outpatients with COVID-19. The loading dose was well tolerated with no discontinuations in this first human use of an 8 g per day initiation regimen of IPE. While the difference in change in hs-CRP between groups was not statistically significant, changes in inflammatory biomarker levels were associated with a significant improvement in patient-reported symptoms over a 14+3-day period. As we await additional larger studies of IPE in the prevention and treatment of COVID-19 (MITIGATE [ClinicalTrials.gov Identifier: NCT04505098] and PREPARE-IT [ClinicalTrials.gov Identifier: NCT04460651]), the results of this preliminary study suggest that IPE may support a safe, well-tolerated, and relatively inexpensive option to manage COVID-19-related symptomatology in the outpatient setting.

### Limitations of the study

Limitations of the study include the modest sample size and open-label design. The trial was not powered for clinical events such as hospitalization or disease progression. Furthermore, the trial only enrolled ambulatory patients and thus commentary on the potential efficacy of IPE use in more advanced, hospitalized patients or its potential role in the prevention of COVID-19 infection is not possible. Moreover, the usage of post hoc statistical analyses is a limitation based on the potential risk of acquiring false positive results.

## STAR★Methods

### Key resources table

**Table:** 

REAGENT or RESOURCE	SOURCE	IDENTIFIER
**Software and algorithms**		

SAS® Software, Version 9.4 (Cary, North Carolina)	SAS Institute Inc.	N/A

**Other**		

InFLUenza Patient-Reported Outcome (FLU-PRO) Diary	Leidos Biomedical Research, Inc.	N/A
Certified Patient Biological Sample Processing Service	Alpha Laboratories Inc.	N/A

### Resource availability

#### Lead contact

Further information and requests for resources should be directed to the corresponding author/lead contact, Dr. Deepak L. Bhatt (dlbhattmd@post.harvard.edu).

#### Materials availability

This study did not generate new unique reagents.

### Experimental model and subject details

#### Study conduct and ethics

This was an investigator-initiated trial, supported by grants from Amarin Pharma Inc. and HLS Therapeutics Inc. to the Canadian Medical and Surgical Knowledge Translation Research Group (CMS). In addition, Amarin Pharma Inc. provided in-kind assistance with the statistical analyses. IPE was provided by HLS Therapeutics Inc. The steering committee was responsible for the development of the protocol, overseeing the conduct of the trial, and interpretation of the results. The study received Health Canada approval on May 8, 2020. The protocol and study materials were approved by the Advarra Institutional Review Board (registered with Office for Human Research Protections [OHRP] and United States Food and Drug Administration [FDA] under IRB#00000971) on May 15, 2020. ClinicalTrials.gov Identifier: NCT04412018. Research members underwent regulatory training, personal protective equipment was worn at all times when in the presence of patients and biohazardous substances, and sensitive files were encrypted and securely stored. Enrolled patients provided verbal and written consent. Sufficient time to read, comprehend and inquire about the study/protocol was provided. IPE capsules were stored appropriately to avoid photo-oxidation and ensure temperature regulation.

#### Study design and population

The VASCEPA-COVID-19 trial was an open-label, prospective, randomized study that enrolled 100 patients to one of two groups. Adults between the ages of 18 and 75 years were eligible to participate in the trial if they met the following inclusion and exclusion criteria. Inclusion criteria: outpatients who have received a positive local SARS-CoV-2 test result within the preceding 72 hours and at least one of the following symptoms: fever, cough, sore throat, shortness of breath, myalgia. Exclusion criteria: individuals participating in another interventional trial that would or may have interfered with the primary outcome, hospitalized individuals, individuals who had a current medical condition for which life expectancy was less than 3 months, individuals with a history of acute end-organ injury (e.g., myocardial infarction, stroke, hospitalization for acute lung, liver, or kidney disease) within the last month, individuals with active severe liver disease, individuals with a history of acute or chronic pancreatitis, women who were pregnant, may be pregnant, were planning on becoming pregnant, or were lactating, women of child-bearing potential who were not using at least one form of highly effective (hormonal contraceptives [e.g., combined oral contraceptives, patch, vaginal ring, injectables, and implants]; intrauterine device or intrauterine system; tubal ligation or whose partner had a vasectomy) and one effective (barrier methods such male condom, female condom, cervical cap, diaphragm, or contraceptive sponge) method of contraception, individuals with a history of hemodynamic instability within the past 72 hours including a systolic blood pressure of <95 mmHg and/or a diastolic blood pressure of <50 mmHg, individuals with known hypersensitivity to fish and/or shellfish, or ingredients of IPE, individuals with any other condition which, in the opinion of the investigator, would place the participant at increased risk, preclude obtaining voluntary consent or confound the objectives of study, individuals who were unable to swallow IPE capsules whole.

#### Study endpoints/outcome measures

The prespecified primary biomarker endpoint was the unadjusted change in high-sensitivity C-reactive protein (hs-CRP) levels from baseline (day 1) to follow-up (day 14 + 3). D-dimer, erythrocyte sedimentation rate (ESR), complete blood count, differential count, serum albumin levels, the neutrophil-to-lymphocyte ratio (NLR), and the systemic immune-inflammation index (defined by platelet count multiplied by NLR) from baseline to follow-up were secondary biomarker endpoints. The prespecified clinical endpoint was the change in InFLUenza Patient-Reported Outcome (FLU-PRO) diary scores (total and domain breakdown). The FLU-PRO score is designed to evaluate the presence, severity, and duration of influenza symptoms in clinical trials and standardize symptom assessment with respect to viral infections (Han et al., 2018; Powers et al., 2018). The diary provides a comprehensive evaluation of symptomology over 32 distinct questions. Respondents answer on an ordinal scale from 0-4 across six domains: Body/Systemic, Chest/Respiratory, Eyes, Gastrointestinal, Nose and Throat. The validity and reliability of the FLU-PRO Diary has been investigated (Han et al., 2018; Powers et al., 2018). The ability to successfully adapt the measure for COVID-19 experimentation has also been explained (https://www.evidera.com/flu-pro/). Two COVID-19-related questions were added (“Do you have loss of taste?”, “Do you have loss of smell?”) to the master questionnaire with yes/no responses and treated as independent domains. Additional clinical endpoints included measuring FLU-PRO symptom prevalence (post hoc), investigating correlations between improvements in FLU-PRO and decreases in hs-CRP levels (post hoc) and assessing a modified World Health Organization (WHO) Symptom Severity Rating (prespecified) (Table S6).

### Method details

#### Sample collection

Eligible patients were randomly allocated to the IPE group or the usual care group. Randomization was performed using envelope randomization via stratification using random permuted blocks. Patients allocated to the IPE group received a loading dose of 4g IPE twice daily for 3 days followed by 2g twice daily for 11 days. The usual care group received no intervention. Patient characteristics (Table S1) were obtained at baseline, while blood samples and clinical outcomes were obtained/measured at baseline and at follow-up. All biological samples were sent to a local certified laboratory for processing.

### Quantification and statistical analysis

#### Number of patients (n) per analysis

Although a total of 100 patients were enrolled in the trial, depending on the availability of the data collected, some analyses included less than 50 individuals per group. For instance, biomarker data were not available for some individuals who had available clinical data. Specifically, Tables 1 and S2–S4 included 44 and 47 patients in the IPE and usual care cohorts, respectively, which removed patients that did not have paired biomarker data and those who were non-compliant to the treatment. Figures 3A and 3B included 46 and 49 patients in the IPE and usual care cohorts, respectively, which removed patients that did not have paired FLU-PRO data and those who were non-compliant to the treatment. Figure 2 and Tables S1, S5 and S6 included the intention-to-treat population (50 individuals in each group), as all data were available for the listed parameters, unless otherwise specified within the figure/table or figure/table legend.

#### Statistical analyses

Baseline characteristics, biomarker, and clinical endpoints were described as frequencies and percentages (for categorical data) or medians with interquartile ranges (for continuous variables). Cohort comparisons were performed with the Wilcoxon Signed Rank (within-group), Mann-Whitney U (between-group, continuous variables) or Fisher’s Exact (between-group, categorical variables) statistical tests. Within- and between-group P-values describing clinical (FLU-PRO) score changes were conducted using a Wald test on least squares-mean estimated treatment difference, from an analysis of variance (ANOVA) model. Within- and between-group comparisons for the primary biomarker endpoint included median change from baseline. hs-CRP analyses were conducted with unadjusted (prespecified) and adjusted (post hoc) data (for sex, age [men <45 versus ≥45 years and women <55 versus ≥55 years], and baseline cardiovascular risk [absence or presence of cardiovascular comorbidities]). The post hoc model adjustments for baseline covariates, which are known to affect inflammation and hs-CRP levels, were warranted based on current literature (O’Driscoll et al., 2020). Remaining unadjusted secondary biomarker data were evaluated via two-tailed within-group Wilcoxon Signed Rank Tests and between-group Mann-Whitney Tests. The prevalence and change from baseline total and individual domain FLU-PRO scores were calculated as measures of alterations in symptom severity. Correlations between FLU-PRO score improvement and hs-CRP reduction were calculated using the Spearman correlation coefficient. A P-value of less than 0.05 was considered significant without multiplicity adjustment and all analyses were conducted using a modified intention-to-treat model. The type of statistical tests used to conduct within- and between-group comparisons were determined post hoc. All statistical analyses were conducted using SAS® software, version 9.4 (Cary, North Carolina).

### Additional resources

The VASCEPA-COVID-19 CardioLink-9 Trial has been registered on ClinicalTrials.gov (Identifier: NCT04412018, URL: https://clinicaltrials.gov/ct2/show/NCT04412018).

## Data Availability

• The patient data reported in this study cannot be deposited in a public repository because of patient privacy and confidentiality. To submit a request for potential access, contact the Corresponding Author/Lead Contact: Deepak L. Bhatt, MD MPH, Brigham and Women’s Hospital Heart and Vascular Center, Harvard Medical School, 75 Francis St., Boston, MA 02115, USA. DLBhattMD@post.Harvard.edu. ORCID: 0000-0002-1278-6245. • This paper does not report original code. • Any additional information required to reanalyze the data reported in this paper is available from the Corresponding Author/Lead Contact upon reasonable request.
