# Supplementary material for: A randomized trial of icosapent ethyl in ambulatory patients with COVID-19
Source: iScience. 2021 Aug 26;24(9):103040. doi: 10.1016/j.isci.2021.103040 (PMC8388138; doi:10.1016/j.isci.2021.103040)
Supplement: Document S1. Tables S1–S6 [file mmc1.pdf]

iScience, Volume *24*

Supplemental information

A randomized trial of icosapent ethyl

in ambulatory patients with COVID-19

Andrew Kosmopoulos, Deepak L. Bhatt, Gus Meglis, Raj Verma, Yi Pan, Adrian Quan, Hwee Teoh, Maya Verma, Lixia Jiao, Robert Wang, Rebecca A. Juliano, Mahesh Kajil, Mikhail N. Kosiborod, Basel Bari, Abdullahi A. Berih, Mallory Aguilar, Antonnette Escano, Andrew Leung, Idelta Coelho, Makoto Hibino, Rafael Díaz, R. Preston Mason, Ph. Gabriel Steg, Tabassome Simon, Alan S. Go, Andrew P. Ambrosy, Richard Choi, Arthur M. Kushner, Lawrence A. Leiter, Mohammed Al-Omran, Subodh Verma, and C. David Mazer

Baseline Characteristics	Icosapent Ethyl (n=50)	Usual Care (n=50)	P-value[a]
Age, Years			
Median (IQR)	46.0 (32.0, 54.0)	40.0 (29.0, 53.0)	0.27
Sex, no. (%)			
Female	26 (52.0)	29 (58.0)	0.69
Male	24 (48.0)	21 (42.0)	0.69
Body Mass Index[b], kg/m^2			
Median (IQR)	24.8 (22.6, 27.1)	24.2 (21.6, 27.3)	0.44
Any comorbidity, no. (%)	18 (36.0)	21 (42.0)	0.68
CAD	1 (2.0)	1 (2.0)	1.00
Cerebrovascular Disease	0 (0)	2 (4.0)	0.49
Diabetes	4 (8.0)	5 (10.0)	1.00
Dyslipidemia	3 (6.0)	7 (14.0)	0.29
Heart Failure	0 (0)	1 (2.0)	1.00
Hypertension	10 (20.0)	7 (14.0)	0.21
Nephropathy	2 (4.0)	0 (0)	0.21
Neuropathy	1 (2.0)	0 (0)	0.46
Peripheral Artery Disease	0 (0)	0 (0)	-
Stroke/TIA	0 (0)	0 (0)	-
Unstable angina	0 (0)	0 (0)	-
Smoker			
Current	0 (0)	3 (6.0)	0.23
Past	0 (0)	1 (2.0)	1.00
Family History of CAD	6 (12.0)	3 (6.0)	0.26
Other	2 (4.0)	2 (4.0)	1.00
Temperature, °C, median (IQR)[c]	36.8 (36.6, 37.0)	36.7 (36.6, 36.9)	0.07
Heart Rate, bpm, median (IQR)[d]	80.0 (71.0, 87.0)	80.0 (74.0, 88.0)	0.54
Sitting Blood Pressure, mmHg[d]			
Systolic, median (IQR)	121.0 (115.0, 129.0)	118.0 (113.0, 131.0)	0.80
Diastolic, median (IQR)	80.0 (77.0, 84.0)	79.0 (75.0, 84.0)	0.21
Lipid Assessment, mmol/L, median (IQR):			
Total Cholesterol[e]	4.2 (3.7, 4.6)	4.3 (3.7, 5.2)	0.17
HDL-C[e]	1.1 (0.9, 1.3)	1.2 (1.0, 1.4)	0.13
LDL-C[f]	2.2 (1.8, 2.6)	2.3 (1.8, 3.0)	0.33
Non-HDL-C[e]	3.2 (2.4, 3.4)	3.0 (2.4, 4.1)	0.45
Triglycerides[e]	1.5 (1.0, 2.4)	1.4 (1.1, 2.3)	1.00

Table S1. Baseline Characteristics, related to STAR METHODS

A list of patient baseline characteristics and associated between-group comparisons listed for the intention-to-treat population. No significant difference in characteristics appeared between the two groups at baseline. The total number of women (n=55) was slightly higher than men (n=45). Comorbidities existed in less than half of the total population. No notable differences in other characteristics such as vital sign measurements and lipid assessments were present. [a]Between-group P-value, calculated via two-tailed Mann-Whitney U Test for continuous variables, Fisher's Exact test for categorical variables. Subjects with missing values were excluded: [b], n=49 in IPE Cohort, n=49 in Usual Care Cohort. [c], n=46 in IPE Cohort, n=49 in Usual Care Cohort. [d], n=45 in IPE Cohort, n=49 in Usual Care Cohort. [e], n=49 in IPE Cohort, n=48 in Usual Care Cohort. [f], n=48 in IPE Cohort, n=46 in Usual Care Cohort. CAD, coronary artery disease; HDL-C, high-density lipoprotein cholesterol; hs-CRP, high-sensitivity C-reactive protein; IQR, interquartile range; LDL-C, low-density lipoprotein cholesterol; TIA, transient ischemic attack.

D-dimer	Baseline (µg/L)	Day 14+3 (µg/L)	P-value (within-group)
Icosapent Ethyl (n=44)	324.0	286.5	0.048
Usual Care (n=47)	292.5	270.0	0.53

P-value (between-groups) = 0.30

Erythrocyte Sedimentation Rate	Baseline (mm/hour)	Day 14+3 (mm/hour)	P-value (within-group)
Icosapent Ethyl (n=44)	18.0	15.5	0.20
Usual Care (n=47)	14.0	15.0	0.96

P-value (between-groups) = 0.28

Table S2. D-dimer and Erythrocyte Sedimentation Rate Biomarker Endpoints, related to STAR METHODS
Median changes in D-dimer and erythrocyte sedimentation rate from baseline to follow-up. A significant within-group D-dimer difference occurred in the IPE cohort (P=0.048) but not within the usual care cohort (P=0.53). The between-group D-dimer comparison was not significant. There were no significant within-group or between-group changes in erythrocyte sedimentation rate. Data are presented as median values.

Secondary Biomarker Endpoints (median)	Icosapent Ethyl (n=44)			Usual Care (n=47)			Absolute Change
	Baseline	14+3 Days	P-value[a]	Baseline	14+3 Days	P-value[a]	P-value[b]
ESR, mm/hr	18.0	15.5	0.20	14.0	15.0	0.96	0.28
D-dimer, µg/L	324.0	286.5	<0.05	292.5	270.0	0.53	0.30
Complete Blood Count							
Hemoglobin, g/L	139.5	130.5	<0.01	144.0	137.0	<0.01	0.66
Hematocrit, L/L	0.4	0.4	<0.01	0.4	0.4	<0.01	0.46
MCV, fl	86.5	87.5	0.03	89.0	89.0	0.32	0.31
MCH, pg	30.0	29.4	0.40	30.0	30.0	0.28	0.22
MCHC, g/L	338.0	339.5	0.22	339.0	338.0	0.31	0.72
RDW, %CV	12.3	12.5	0.14	12.2	12.2	0.62	0.37
Platelet, $*10^9$/L	227.0	261.5	<0.01	235.0	253.0	0.13	0.24
RBC Count, $*10^{12}$/L	4.9	4.7	<0.01	4.7	4.5	<0.01	0.96
WBC Count, $*10^9$/L	6.1	7.3	<0.01	6.0	6.8	0.01	0.04
WBC Differential, $*10^9$/L							
Neutrophils	3.0	3.9	<0.01	3.4	3.6	0.10	0.02
Lymphocytes	1.9	2.4	0.01	2.1	2.1	0.06	0.32
Monocytes	0.5	0.6	0.08	0.5	0.5	0.23	0.66
Eosinophils	0.1	0.1	0.07	0.1	0.1	<0.01	0.56
Basophils	0.0	0.0	0.07	0.0	0.0	0.69	0.24
NLR	1.7	1.6	0.07	1.6	1.6	0.45	0.25
SII, $*10^9$/L	339.8	431.9	<0.01	427.0	411.7	0.26	0.06
HbA1C, %	5.2	5.1	<0.01	5.2	5.2	0.01	0.37
Albumin, g/L	44.0	43.0	0.88	45.0	44.0	<0.01	0.03
Creatinine, µmol/L	74.0	73.5	0.21	67.0	71.0	0.46	0.12
eGFR, mL/min/1.73m^2	86.0	87.0	0.17	92.0	90.0	0.30	0.06
Lipid Assessment, mmol/L							
Total Cholesterol	4.3	4.7	<0.01	4.3	4.7	0.17	0.09
HDL-C	1.1	1.2	<0.01	1.2	1.3	0.14	0.08
LDL-C	2.2	2.5	<0.01	2.3	2.5	0.04	0.32
Non-HDL-C	3.2	3.3	<0.01	3.0	3.3	0.29	0.24
Triglycerides	1.4	1.5	0.19	1.4	1.2	0.04	0.52

Table S3. Secondary Biomarker Endpoints, related to STAR METHODS
Within-group comparisons of secondary biomarker endpoints. D-dimer was significantly reduced within the IPE group but not within the usual care group. The IPE group experienced a statistically significant rise in platelets that was not observed in the usual care group, leading to a significant increase in the systemic immune-inflammation index (SII) within the IPE group. SII was calculated as the product of NLR*platelets. Patients with paired blood samples were included in the analyses. [a]Within-group P-value, calculated via two-tailed Wilcoxon Sign Rank Test. [b]Between-group P-value, calculated via Mann-Whitney U Test. eGFR, estimated glomerular filtration rate; ESR, erythrocyte sedimentation rate; HbA1C, hemoglobin A1c; HDL-C, high-density lipoprotein cholesterol; LDL-C, low-density lipoprotein cholesterol; MCH, mean corpuscular hemoglobin; MCHC, mean corpuscular hemoglobin concentration; MCV, mean corpuscular volume; NLR, neutrophil-lymphocyte ratio; Non-HDL-C, non-high-density lipoprotein cholesterol; RBC, red blood cell; RDW, red cell distribution width; WBC, white blood cell.

FLU-PRO Scores	**Icosapent Ethyl (n=44)**		**Usual Care (n=47)**	
	Spearman Correlation Coefficient	P-value	Spearman Correlation Coefficient	P-value
Total	41%	0.005	22%	0.14
Body/Systemic	41%	0.006	18%	0.24
Chest/Respiratory	57%	<0.001	3%	0.86

Table S4. Post hoc Correlation Analysis Between FLU-PRO Score and hs-CRP Levels, related to STAR METHODS
Correlations of FLU-PRO scores compared to hs-CRP levels. Significant correlations were found between the improvement (reduction) in FLU-PRO scores (in the total [P=0.005], body/systemic [P=0.006] and chest/respiratory [P<0.001] domains) and the decrease in hs-CRP levels within the IPE arm. Significant correlations were not seen in the usual care arm. FLU-PRO, InFLUenza Patient-Reported Outcome; hs-CRP, high-sensitivity C-reactive protein.

n (%)	Icosapent Ethyl (n=50)	Usual Care (n=50)
Total adverse events	6 (12)	3 (6)
Mild adverse events		
Gastrointestinal disorders	4 (8)	0 (0)
Moderate adverse events		
Gastrointestinal disorders	1 (2)	0 (0)
Respiratory disorders	1 (2)	3 (6)

Table S5. Adverse Events, related to STAR METHODS
Total adverse events (AEs) listed for the intention-to-treat population. The relationship between mild gastrointestinal disorders and IPE is unclear. Moderate AEs resulted in hospitalization which were not related to the investigational product in the treatment group. No AEs were deemed a result of the IPE loading dose. The three moderate AEs in the usual care group resulted in same day emergency room visits. These patients were released with steroids (n=1), antibiotics (n=1) or no treatment (n=1). No serious AEs or deaths occurred in the trial population.

Visit/WHO SS Rating, n(%)	Icosapent Ethyl (n=50)			Usual Care (n=50)		
	WHO SS Rating 1	WHO SS Rating 2	Missing Rating	WHO SS Rating 1	WHO SS Rating 2	Missing Rating
Baseline	18 (36)	32 (64)	0	27 (54)	23 (46)	0
Day 14+3	47 (94)	1 (2)	2 (4)	47 (94)	2 (4)	1 (2)

Table S6. Modified World Health Organization (WHO) Symptom Severity Rating, related to STAR METHODS
A summary of the modified WHO Symptom Severity Ratings per group listed for the intention-to-treat population. A rating of 1 corresponds to non-hospitalized patients who were able to perform normal activities. A rating of 2 corresponds to non-hospitalized patients who were unable to perform normal activities. All patients started on the low end of the severity scale (i.e., with a score of 1 or 2 from a range of 1-7) and almost all resolved to a score of 1 at Day 14+3, with the exception of 1 patient in IPE arm and 2 patients in usual care arm. The lowest possible score was 1, incrementally increasing in severity to the highest possible score of 7; a score 3-6 indicates hospitalization; a score of 7 indicates death. SS, Symptom Severity.
